# Roles and Prospective Applications of Ferroptosis Suppressor Protein 1 (FSP1) in Malignant Tumor Treatment

**DOI:** 10.3390/curroncol32080456

**Published:** 2025-08-14

**Authors:** Zhesi Jin, Qian Zhang, Yinlong Pan, Hao Chen, Ke Zhou, Huazhong Cai, Pan Huang

**Affiliations:** School of Medicine, Jiangsu University, Zhenjiang 212013, China; 2222313134@stmail.ujs.edu.cn (Z.J.); 2222313015@stmail.ujs.edu.cn (Q.Z.); 2222213137@stmail.ujs.edu.cn (Y.P.); 2222313127@stmail.ujs.edu.cn (H.C.); 2212413146@stmail.ujs.edu.cn (K.Z.)

**Keywords:** FSP1, ferroptosis, tumor, CoQ10, targeted therapy

## Abstract

Ferroptosis suppressor protein 1 (FSP1) has emerged as a critical regulator of ferroptosis, an iron-dependent form of programmed cell death with significant therapeutic potential in cancer treatment. Despite rapidly expanding research, current knowledge on FSP1 remains fragmented across various tumor types and experimental contexts. The aim of this review is to systematically integrate the latest evidence regarding the molecular structure, biological functions, and regulatory mechanisms controlling FSP1 expression, emphasizing its involvement in tumor progression and resistance to therapy. Readers can expect comprehensive coverage of FSP1’s structural characteristics, enzymatic roles, transcriptional and post-transcriptional regulation, and its pathological significance in hepatocellular carcinoma, colorectal cancer, pancreatic cancer, gastric cancer, breast cancer, lung cancer, and leukemia. We further evaluate emerging therapeutic strategies targeting FSP1 aimed at overcoming resistance and improving clinical outcomes. Relevant studies were systematically identified by searching PubMed, Web of Science, and Embase databases, focusing particularly on the recent and impactful literature to guide future research directions.

## Simple Summary

Many cancer treatments fail because tumor cells evade ferroptosis, a type of cell death caused by iron-dependent damage. A critical protector of tumor cells against ferroptosis is ferroptosis suppressor protein 1 (FSP1), which repairs lipid damage that would otherwise kill these cells. While research into FSP1 is rapidly expanding, current findings remain fragmented across different cancer types, leaving key questions unanswered about how FSP1 becomes activated, its role in cancer progression, and how best to therapeutically target it. This review consolidates existing evidence, highlighting that elevated FSP1 often marks aggressive forms of liver, colorectal, pancreatic, gastric, breast, lung, and blood cancers. We also discuss new drugs and treatment combinations designed to inhibit FSP1. By clarifying what is known and identifying knowledge gaps, this article provides researchers and clinicians with a foundation to develop therapies that overcome treatment resistance and improve patient outcomes.

## 1. Introduction

Ferroptosis is a regulated form of cell death characterized by abnormal iron accumulation and disrupted redox balance, which ultimately leads to extensive lipid peroxidation [1]. The consequent imbalance within the intracellular antioxidant defense system leads to the disruption of the cell membrane and, ultimately, cell death [1,2,3,4]. Morphologically, cells undergoing ferroptosis are characterized by smaller mitochondria exhibiting increased membrane density, disrupted outer membranes, and markedly diminished or absent cristae [1]. These unique characteristics set ferroptosis apart from other cell death modalities, including apoptosis, necrosis, and autophagy [1,5]. In cancer treatment, it is critical to selectively target tumor cells while preserving normal cells [6]. The former, owing to defective cell death execution mechanisms and increased iron requirement, are more prone to iron-catalyzed oxidative damage, rendering them particularly vulnerable to ferroptosis [6]. By promoting iron overload and lipid radical accumulation, ferroptosis selectively eradicates tumor cells and effectively synergizes with conventional therapies such as radiotherapy and chemotherapy, amplifying oxidative stress and overcoming tumor defense barriers [2,5,7,8].

Glutathione peroxidase 4 (GPX4), a core regulator of ferroptosis, directly triggers irreversible tumor cell death upon loss of its activity, providing a theoretical foundation for ferroptosis-based cancer therapies [9]. However, certain tumor cell lines remain resistant to ferroptosis despite complete GPX4 inhibition, suggesting the existence of compensatory regulatory mechanisms independent of GPX4 [10]. Accumulating evidence indicates that GPX4-resistant cancer cells heavily rely on ferroptosis suppressor protein 1 (FSP1), which acts as a critical node integrating multiple protective pathways [7,11]. Recent studies have demonstrated that FSP1 utilizes nicotinamide adenine dinucleotide (phosphate) (NAD(P)H) to reduce oxidized coenzyme Q10 (CoQ10) to ubiquinol (CoQ10H_2_), functioning in parallel with the glutathione (GSH)/GPX4 pathway to inhibit phospholipid peroxidation, thus establishing a coordinated antioxidant defense system [11]. Furthermore, FSP1 catalyzes the reduction of vitamin K (VK) to vitamin K hydroquinone (VKH_2_), a potent antioxidant that significantly enhances ferroptosis resistance by protecting cancer cells from iron-mediated oxidative damage [12,13,14]. Consequently, simultaneous targeting of FSP1 and its associated antioxidant defense mechanisms could comprehensively dismantle these redundant protective barriers, effectively overcoming ferroptosis resistance [10]. In this review, we comprehensively discuss the structural and functional characteristics of FSP1, elucidate its regulatory mechanisms, and systematically summarize its roles across multiple malignancies. Additionally, we outline current FSP1-targeted therapeutic strategies, existing challenges, and potential future research directions, providing valuable theoretical insights and innovative perspectives to advance anticancer therapies targeting FSP1.

## 2. Structure and Function of FSP1

The human FSP1 gene, located on chromosome 10q21.3–q22.1, encodes a protein consisting of 373 amino acids with an approximate molecular mass of 40.5 kDa [15]. FSP1 functions as an NAD(P)H-dependent oxidoreductase primarily localized to the plasma membrane and various other non-mitochondrial membranes [16,17,18]. Structurally, FSP1 possesses critical domains for NAD(P)H and flavin adenine dinucleotide (FAD) binding, a distinctive C-terminal region, and an essential N-terminal myristoylation motif for membrane localization [11,18,19,20]. Additionally, FSP1 contains intrinsically disordered regions (IDRs) capable of driving liquid–liquid phase separation (LLPS), resulting in functional condensate formation at damaged membrane sites. This mechanism facilitates rapid local scavenging of lipid peroxyl radicals and membrane repair [11,21,22,23,24]. Accumulating evidence indicates that elevated FSP1 expression not only counteracts canonical ferroptosis inducers but also drives resistance to frontline chemotherapeutics across multiple tumour types. Mechanistically, FSP1 acts as an NAD(P)H–CoQ_10_ oxidoreductase that regenerates ubiquinol and vitamin K hydroquinone, intercepting phospholipid peroxyl radicals and suppressing lipid-ROS-triggered ferroptosis [11]. In colorectal cancer, FSP1 cooperates with GPX4 to uphold an EMT programme and shields cells from 5-fluorouracil (5-FU). In pancreatic ductal adenocarcinoma, Snail-driven FSP1 upregulation underlies resistance to gemcitabine. In ovarian cancer, an NRF2–JAM3–FSP1 axis mitigates lipid peroxidation and mediates cross-resistance to cisplatin and PARP inhibitors. Elevated FSP1 likewise limits the efficacy of sorafenib in hepatocellular carcinoma and of abiraterone in metastatic prostate cancer [25,26]. Genetic or pharmacological ablation of FSP1 restores ferroptotic vulnerability and resensitizes tumours to these agents (see Section 4 for cancer-specific details). Novel FSP1 inhibitors, such as improved chemical inhibitor of ferroptosis suppressor protein 1 (icFSP1), disrupt LLPS, impairing membrane localization and dimerization, thereby sensitizing cancer cells to ferroptosis [23]. Collectively, these structural characteristics and LLPS properties underscore the therapeutic potential of targeting FSP1 to overcome tumor drug resistance.

## 3. Regulation of FSP1 Expression

**Figure 1:**
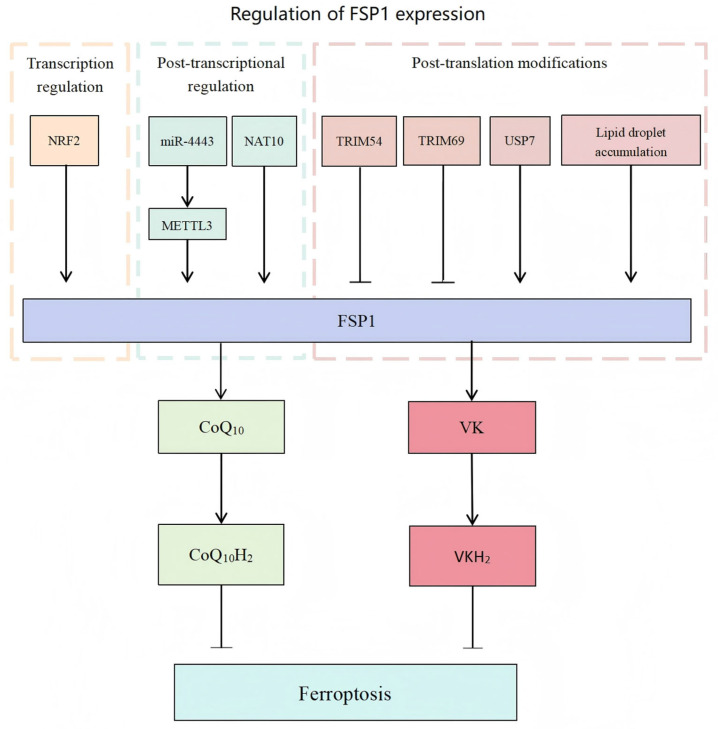
FSP1 inhibits ferroptosis by reducing CoQ10 and vitamin K to their antioxidant forms, CoQ10H_2_ and VKH_2_, respectively. Its expression is regulated at three levels: (1) transcriptionally by NRF2 via ARE binding; (2) post-transcriptionally by miR-4443 (inhibiting m^6^A via METTL3) and NAT10 (enhancing mRNA stability via ac^4^C); (3) post-translationally by TRIM54/TRIM69-mediated ubiquitination, USP7-mediated deubiquitination, and lipid droplet accumulation. These layers converge to enhance ferroptosis resistance across multiple cancers.

The expression and activity of FSP1 are precisely regulated via diverse mechanisms at the transcriptional, post-transcriptional, and post-translational stages, collectively influencing its critical role in maintaining cellular redox balance. A schematic overview of the multi-layered regulatory network controlling FSP1 expression is provided in Figure 1.

### 3.1. Transcriptional Regulation

The oxidative stress-responsive nuclear factor erythroid 2-related factor 2 (NRF2)/Kelch-like ECH-associated protein 1 (KEAP1) signaling axis functions as an essential upstream modulator of FSP1 transcription [7]. Under homeostatic conditions, NRF2 remains bound to its inhibitor, KEAP1, and undergoes continuous degradation [27]. In response to oxidative stress or the mutation-induced oxidative inactivation of KEAP1, NRF2 dissociates from KEAP1-mediated degradation, translocates to the nucleus, and activates the transcription of various antioxidant genes, including FSP1, by binding to antioxidant response elements (AREs) [28,29]. FSP1 has been confirmed as a direct transcriptional target of NRF2; notably, in KEAP1-deficient lung cancer cells, FSP1 is among the most highly upregulated genes driven by NRF2, whereas NRF2 knockout significantly reduces FSP1 expression and increases cellular susceptibility to ferroptosis [27].

### 3.2. Post-Transcriptional Regulation

FSP1 expression is also subject to post-transcriptional regulation, particularly through messenger RNA (mRNA) modifications, such as N6-methyladenosine (m6A) and N4-acetylcytidine (ac4C) [30]. m6A modification regulates FSP1 expression by modulating mRNA stability and translational efficiency [31]. For instance, in lung cancer cells, the m6A methyltransferase methyltransferase-like 3 (METTL3) catalyzes the m6A modification of FSP1 mRNA, which is subsequently recognized by the m6A reader protein YTH Domain Family Member 2 (YTHDF2). This interaction leads to decreased mRNA stability and lower levels of FSP1 [32]. Conversely, the loss of another m6A reader protein, YTH Domain Containing 1 (YTHDC1), significantly elevates FSP1 expression [33,34]. Additionally, tumor-derived exosomal microRNA-4443 (miR-4443) indirectly upregulates this expression by downregulating METTL3, thus decreasing m6A modification on FSP1 mRNA. Elevated FSP1 expression enhances cellular antioxidant capacity, reducing lipid peroxidation and promoting resistance to ferroptosis-induced cell death. This mechanism has been demonstrated to significantly contribute to cisplatin resistance in non-small-cell lung cancer (NSCLC) cells [31,35,36]. In colorectal cancer (CRC), the RNA acetyltransferase N-acetyltransferase 10 (NAT10) is frequently upregulated [30]. NAT10 catalyzes N4-acetylcytidine (ac4C) modification at specific sites on FSP1 mRNA, enhancing its stability and translation and ultimately leading to increased FSP1 expression [37]. This elevation in FSP1 strengthens cancer cell resistance to ferroptosis and promotes tumor progression [37]. In addition, long non-coding RNAs (lncRNAs) and circular RNAs (circRNAs) are involved in regulatory processes at the post-transcriptional level [38].

### 3.3. Post-Translational Modifications

The function of FSP1 is finely regulated by post-translational modifications, including ubiquitination and acetylation, which influence its stability, localization, and biological activity [26,38]. Regarding ubiquitination, the E3 ubiquitin ligase tripartite motif containing 21 (TRIM21) mediates K63-linked ubiquitination of FSP1, facilitating its translocation to the plasma membrane, where it exerts potent anti-lipid peroxidation activity, thus enhancing ferroptosis resistance [39]. Conversely, in hepatocellular carcinoma (HCC), sorafenib activates the extracellular signal-regulated kinase (ERK) signaling pathway, promoting tripartite motif containing 54 (TRIM54)-mediated ubiquitination and proteasomal degradation of FSP1, thereby reducing FSP1 protein abundance and sensitizing tumor cells to ferroptosis [26]. Similarly, tripartite motif containing 69, another E3 ubiquitin ligase, directly promotes ubiquitination-dependent degradation of FSP1 in HCC cells. Notably, this TRIM69-mediated ubiquitination can be competitively blocked by ferroptosis-associated long non-coding RNA (lncFAL), which binds directly to FSP1, thus stabilizing the FSP1 protein and ultimately enhancing ferroptosis resistance [38]. In gastric cancer (GC), lipid droplet accumulation correlates with decreased ubiquitination and enhanced stabilization of FSP1, leading to increased ferroptosis resistance [40]. In melanoma, the deubiquitinase ubiquitin-specific protease 7 (USP7) indirectly enhances FSP1 expression and ferroptosis resistance through stabilization of the transcription factor Jun D proto-oncogene (JunD) [41].

Furthermore, the acetylation level of FSP1 is regulated by cellular metabolic status, particularly by the availability of cytosolic acetyl-coenzyme A (acetyl-CoA) [42]. The mitochondrial citrate carrier solute carrier family 25 member 1 (SLC25A1) transports citrate from mitochondria into the cytoplasm, where ATP-citrate lyase (ACLY) converts it into acetyl-CoA [42]. Under conditions of abundant cytosolic acetyl-CoA, an acetyltransferase acetylates FSP1 at Lys127, blocking β-transducin repeat-containing protein (β-TrCP) binding and preventing K48-linked ubiquitination and proteasomal degradation, thus significantly extending the half-life of FSP1 [42]. The knockdown of SLC25A1 or ACLY depletes cytosolic acetyl-CoA, reduces FSP1 stability, and enhances cellular sensitivity to ferroptosis, whereas supplementation with exogenous acetate can partially restore acetyl-CoA levels and FSP1 function [42].

## 4. Roles of FSP1 in Different Types of Tumors

### 4.1. Hepatocellular Carcinoma

In hepatocellular carcinoma (HCC), approximately 37% of tumors exhibit elevated FSP1 expression, which significantly correlates with poor clinical outcomes [43]. HCC cells heavily depend on the KEAP1/NRF2-driven FSP1–CoQ_10_ antioxidant pathway to resist ferroptosis [43]. Detailed molecular mechanisms involving TRIM69-mediated ubiquitination and its competitive inhibition by lncFAL are comprehensively discussed in Section 3.2 and Section 3.3, and illustrated in Figure 1. Preclinical studies have demonstrated that HCC xenografts with high FSP1 expression exhibit reduced sensitivity to GPX4 inhibitors; however, simultaneous targeting of FSP1 markedly restores tumor vulnerability, underscoring the therapeutic potential of this pathway.

High-density lipoprotein-binding protein (HDLBP) binds to and stabilizes lncFAL, suggesting that targeting HDLBP or lncFAL may represent effective strategies against HCC with elevated lncFAL expression [38]. In contrast, the multi-kinase inhibitor sorafenib activates the ERK signaling pathway, promoting TRIM54-mediated ubiquitination and the subsequent proteasomal degradation of FSP1, leading to reduced FSP1 protein levels and the induction of ferroptosis in HCC cells [26]. Moreover, circular RNA circ0060467 acts as a molecular sponge for miR-6805, alleviating miRNA-mediated repression at the post-transcriptional level and increasing FSP1 and GPX4 mRNA abundance, thus ultimately enhancing their expression, inhibiting ferroptosis, and promoting tumor growth in HCC [44].

The specific small-molecule inhibitor of ferroptosis suppressor protein 1 (iFSP1) not only effectively induces ferroptosis and reduces tumor burden in HCC models but also significantly reshapes the tumor immune microenvironment via FSP1-dependent mechanisms [43]. By inhibiting FSP1, iFSP1 intensifies lipid peroxidation and ferroptotic stress, leading to enhanced release of damage-associated molecular patterns (DAMPs). These DAMPs promote dendritic cell maturation and antigen presentation, thereby markedly increasing tumor infiltration by dendritic cells, macrophages, and T lymphocytes [43,45]. Thus, FSP1 inhibition via iFSP1 not only directly sensitizes tumor cells to ferroptosis but also indirectly enhances antitumor immune responses, highlighting its potential for combinational therapeutic strategies [6,27]. Additionally, combining an FSP1 inhibitor with immune checkpoint inhibitors demonstrates synergistic effects in suppressing HCC growth, suggesting that targeting FSP1 may enhance the efficacy of immunotherapy [43,46]. Moreover, the mechanistic target of the rapamycin (mTOR) inhibitor, temsirolimus, has been found to directly bind and inhibit FSP1 activity, thereby inducing ferroptosis in liver cancer cells. When used in combination with the GPX4 inhibitor RSL3, temsirolimus effectively suppresses HCC progression [47]. These findings underscore the high dependency of HCC on FSP1, highlighting its potential as a promising therapeutic target [43,46].

In addition to influencing cell survival, the abnormal activation of FSP1 may also facilitate invasion and metastasis in HCC [48]. High FSP1 expression can promote HCC metastasis through pathways independent of ferroptosis [49]. Specifically, FSP1 promotes mitochondrial biogenesis by activating the sirtuin 1 (SIRT1)/peroxisome proliferator-activated receptor gamma coactivator 1-alpha (PGC-1α) axis, thereby enhancing the metabolic activity and invasive capacity of tumor cells [48]. Conversely, knocking down FSP1 significantly inhibits HCC cell migration and the formation of lung metastatic nodules [43,48]. Therefore, in liver cancer, FSP1 not only affects sensitivity to ferroptosis but also promotes malignant progression through mechanisms such as metabolic reprogramming.

### 4.2. Colorectal Cancer

FSP1 expression is markedly upregulated in CRC tissues compared to that in adjacent normal tissues, and this increase is strongly associated with advanced tumor–node–metastasis (TNM) stages, lymph node involvement, distant metastasis, and unfavorable patient outcomes [50]. Mechanistically, FSP1 sustains a low-lipid-ROS milieu that stabilises NRF2; nuclear NRF2 directly upregulates the epithelial–mesenchymal transition (EMT) transcription factors Snail and Slug, leading to E-cadherin loss and Vimentin/N-cadherin induction. Silencing or pharmacological inhibition of FSP1 elevates lipid ROS, reactivates GSK3β-dependent Snail degradation, restores E-cadherin, and markedly suppresses CRC-cell migration [30]. The RNA acetyltransferase NAT10, a key upstream regulator of FSP1 expression, is frequently upregulated in CRC, contributing significantly to elevated FSP1 expression via the previously described ac4C modification mechanism [30]. The upregulated NAT10–FSP1 axis not only strengthens ferroptosis resistance and aggressive phenotypes (proliferation, migration, invasion) but also predicts poor prognosis in CRC [30]. Furthermore, trans-3-indoleacrylic acid (IDA), a metabolite secreted by gut microbiota, activates the aryl hydrocarbon receptor (AHR), upregulating aldehyde dehydrogenase 1A3 (ALDH1A3) and enhancing nicotinamide adenine dinucleotide (NADH) availability [51]. This drives the FSP1-mediated reduction of CoQ10, thereby inhibiting ferroptosis and promoting CRC progression [52].

Given FSP1’s central role in CRC progression and ferroptosis resistance, combined targeting of FSP1 (or its upstream regulator NAT10) and GPX4 has emerged as a superior strategy, more effectively inducing CRC cell death than inhibition of either target alone [30,53]. Furthermore, high levels of GPX4 and FSP1 have been implicated in 5-fluorouracil (5-FU) resistance and metastasis in CRC via EMT-driven mechanisms [54]. Natural compounds such as curcumin and andrographolide, which concurrently downregulate GPX4 and FSP1, not only synergistically induce ferroptosis but also reverse EMT marker expression—upregulating E-cadherin and downregulating Vimentin and N-cadherin—and inhibit CRC cell invasion. In mouse xenograft models, co-administration of these compounds with 5-FU significantly reduces metastatic nodule formation in the liver and lungs, restoring chemosensitivity and preventing tumour dissemination [55,56]. These results highlight the dual therapeutic potential of GPX4/FSP1 co-inhibition for overcoming chemotherapy resistance and suppressing metastasis in CRC.

### 4.3. Pancreatic Ductal Adenocarcinoma (PDAC)

PDAC frequently harbors activating KRAS mutations, which notably enhance FSP1 expression by triggering the mitogen-activated protein kinase (MAPK)/NRF2 signaling cascade [57]. Compared to normal pancreatic tissues, PDAC tissues exhibit elevated FSP1 levels, positively correlating with NRF2 expression in patient tumor specimens [58,59,60]. In KRAS-mutated PDAC cells, FSP1-mediated ferroptosis suppression is crucial for cell survival; specifically, FSP1 overexpression enhances cellular resistance to lipid peroxidation damage, promotes organoid/spheroid formation and growth in three-dimensional culture models, and accelerates tumor initiation and progression [57]. Beyond ferroptosis control, high FSP1 maintains a low-lipid-ROS milieu that stabilizes the EMT transcription factors Snail and Slug, downregulates E-cadherin, and upregulates Vimentin/N-cadherin, thereby driving an epithelial-to-mesenchymal transition (EMT) program that confers intrinsic resistance to gemcitabine [39]. Genetic or pharmacologic inhibition of FSP1 elevates lipid ROS, induces glycogen synthase kinase 3β (GSK3β)-dependent Snail degradation, reverses EMT markers, and resensitizes orthotopic KRAS-G12D PDAC tumors to gemcitabine [61]. In KRAS G12D-driven PDAC models, the sole inhibition of GPX4 results in weak or reversible lipid peroxidation, thereby exerting only limited effects on organoid viability [57]. However, the simultaneous blockade of FSP1-mediated antioxidant defenses dramatically amplifies drug-induced ferroptosis signals, rapidly triggering the collapse and complete inactivation of organoids [13,57]. Taken together, these observations indicate that FSP1 and GPX4 cooperatively establish a dual-layer antioxidant defense system in PDAC cells, enabling KRAS-mutated tumor cells to bypass the “ferroptosis checkpoint” and facilitating their survival and proliferation [27,62,63].

### 4.4. Gastric Cancer

FSP1 exhibits elevated expression levels in GC tissues, which strongly correlate with tumor progression and unfavorable clinical outcomes [64,65]. In GC, FSP1 frequently undergoes coordinated upregulation with the mitochondrial iron–sulfur protein CISD1 (MitoNEET), and the high co-expression of these two proteins predicts notably poorer survival outcomes [64]. Additionally, the combination of low FSP1 expression with high levels of the lipid peroxidation indicator, 4-hydroxynonenal (4-HNE), accumulation is associated with improved patient prognosis; however, when assessed independently, the difference in Kaplan–Meier survival curves based solely on FSP1 expression does not reach statistical significance [64]. This suggests that the prognostic role of FSP1 in gastric cancer may be modulated by multiple factors. Moreover, gastric tumors with high FSP1 expression exhibit abnormal infiltration patterns of CD8^+^ T cells and macrophages, indicating that FSP1 may influence treatment efficacy by reshaping the tumor immune microenvironment [65]. Gastric cancer stem cells (GCSCs) generally display lower ROS levels and higher chemoresistance; thus, co-targeting FSP1 and ferroptosis inducers to disrupt redox homeostasis could effectively reduce tumor recurrence risk [66,67]. In gastric cancer cells, the proteins glycerol-3-phosphate dehydrogenase 1 (GPD1) and glycerol-3-phosphate dehydrogenase 1-like (GPD1L) drive aberrant lipid droplet accumulation, which subsequently elevates FSP1 expression, enhances resistance to ferroptosis, and promotes peritoneal metastasis [40]. The targeted inhibition of GPD1/GPD1L or FSP1 reduces lipid droplet formation, lowers the threshold for ferroptosis resistance, and significantly suppresses metastatic lesion formation [40].

### 4.5. Breast Cancer

FSP1 is expressed at markedly higher levels in breast cancer—especially triple-negative breast cancer (TNBC)—compared with normal breast tissue [68]. Increased FSP1 levels strongly correlate with poor clinical outcomes, including decreased survival rates and an increased risk of metastasis [68], while the inhibition of FSP1 oxidoreductase activity can effectively delay malignant tumor progression [7,68]. TNBC lacks expression of the estrogen receptor, the progesterone receptor, and human epidermal growth factor receptor 2 (HER2), receptors that are critical targets for effective therapies; their absence thus limits treatment options, increasing the risk of therapeutic resistance and recurrence [69]. The aggressive behavior of TNBC is closely linked to FSP1-mediated resistance to ferroptosis [70,71]. Through its CoQ10 oxidoreductase activity, FSP1 maintains the clearance of lipid peroxides, enabling TNBC cells to withstand oxidative stress within the tumor microenvironment (TME) [11]. The unique iron metabolism characteristics of TNBC cells—namely, high intracellular iron content combined with limited antioxidant capacity—render them highly dependent on FSP1 [68]. This protein is critically important for tumor cell survival, particularly under the hypoxic and nutrient-limited conditions characteristic of the TME [7,68].

Elevated FSP1 expression confers resistance to GPX4 inhibitor-induced ferroptosis across multiple cancer types, including TNBC, whereas the genetic ablation of FSP1 markedly sensitizes TNBC cells to ferroptosis-inducing agents [11,68]. Elevated FSP1 expression is also associated with resistance to conventional chemotherapy in TNBC [68]. To overcome this FSP1-mediated ferroptosis resistance, Yang et al. developed a metabolic intervention nanoparticle formulation, encapsulating the statin drug rosuvastatin within copper ion–silk fibroin nanoparticles [68]. On the one hand, this nanotherapeutic approach inhibits the cholesterol/CoQ10 biosynthetic pathway, thereby disrupting the FSP1-dependent CoQ10 antioxidant equilibrium; on the other hand, the formulation releases copper ions to generate ROS and deplete intracellular GSH, thereby destabilizing the redox balance and effectively inducing ferroptosis in TNBC cells [68]. In conclusion, inhibiting FSP1 to induce ferroptosis has emerged as an effective approach for combating therapy-refractory breast cancers, notably the triple-negative subtype.

### 4.6. Lung Cancer

In lung cancer, dysregulated FSP1 expression is closely associated with intricate modulation by multiple signaling pathways [57]. KRAS mutations activate the MAPK-NRF2 axis, leading to the elevated expression of FSP1 and conferring resistance to ferroptosis in tumor cells [57,72,73]. Similarly, inactivating mutations of KEAP1 result in the hyperactivation of NRF2, thereby strongly inducing FSP1 expression and conferring robust resistance to both ferroptosis and radiation-induced damage [74,75]. In KRAS-driven lung adenocarcinoma, concurrent mutations of KEAP1 and liver kinase B1 (LKB1) are common [57]. These co-mutations shape a distinct metabolic–immune phenotype, significantly enhancing ferroptosis tolerance in this particular tumor subtype [76].

Currently, targeted therapies against FSP1 have demonstrated promising efficacy in lung cancer models [74]. Both the first-generation inhibitor iFSP1 and the novel inhibitor icFSP1 significantly enhance ferroptosis induction in lung cancer and other tumor cells [23]. icFSP1 significantly enhances sensitivity to ferroptosis by disrupting FSP1 function via the previously described phase separation mechanism [23]. When combined with GPX4 inhibitors, icFSP1 synergistically triggers a potent ferroptotic response in tumor cells [23,77]. In a lung cancer H460 xenograft model, the simultaneous inhibition of GPX4 and FSP1 was necessary to suppress tumor growth, suggesting that therapies targeting GPX4 alone might fail due to the compensatory activation of FSP1 [11]. Thus, incorporating an FSP1 inhibitor can overcome resistance to GPX4-targeted therapies [11]. Additionally, icFSP1 markedly inhibits tumor growth and elevates the abundance of the lipid peroxidation indicator 4-hydroxynonenal (4-HNE) in melanoma and lung cancer models, demonstrating enhanced metabolic stability and improved efficacy in vivo relative to iFSP1 [39]. Additionally, the natural compound curcumin has been shown to induce ferroptosis in lung cancer stem-like cells via the FSP1–CoQ10–NADH signaling pathway, dose-dependently reducing cell viability and markedly downregulating FSP1 expression [78]. However, research by Takahara et al. in lung adenocarcinoma indicated that low FSP1 expression was significantly associated with poorer recurrence-free survival [27,77]. One plausible explanation is that tumors with intrinsically low FSP1 compensate by upregulating alternative ferroptosis shields such as GPX4, solute carrier family 7 member 11 (SLC7A11), or other NRF2-driven antioxidant enzymes, permitting aggressive growth despite ex vivo ferroptotic vulnerability [73]. In addition, stromal and immune cells may supply exogenous CoQ_10_ or vitamin K, partially rescuing FSP1-deficient cancer cells in vivo. These compensatory inputs could offset the ferroptosis-sensitizing effect of low FSP1, translating into poorer clinical outcomes. Further multi-omics analyses that quantify GPX4/SLC7A11 activity, metabolite availability, and immune-cell infiltration are warranted to validate this hypothesis.

### 4.7. Leukemia

In hematologic malignancies, the regulatory mechanisms of ferroptosis differ significantly from those in solid tumors. Acute lymphoblastic leukemia (ALL) cells primarily rely on the GSH–GPX4 pathway to maintain redox homeostasis, while exhibiting minimal dependence on the FSP1–CoQ10 pathway [79]. In ALL cells, the promoter region of the FSP1 gene is characterized by aberrantly high methylation, an epigenetic alteration that directly results in transcriptional silencing [79]. Markedly reduced expression of FSP1 has been observed in both ALL cell lines and primary patient samples; the exogenous restoration of FSP1 significantly enhances cell survival and effectively inhibits ferroptosis activation [80]. Notably, when the GSH–GPX4 core pathway is compromised, ALL cells rapidly activate ferroptosis due to the absence of compensatory antioxidant defenses mediated by FSP1 [79]. This provides a theoretical foundation for combined therapeutic strategies that target epigenetic regulation alongside ferroptosis induction in the treatment of ALL [79,81].

Acute myeloid leukemia (AML) displays an opposite ferroptotic phenotype compared with ALL, with both GPX4 and FSP1 highly expressed in AML tumor tissues [82]. Several studies show that high-FSP1 AML cell lines (KG-1, U937) and primary FLT3-ITD^+^ blasts survive pharmacologic GPX4 inhibition (RSL3, ML210) but undergo rapid ferroptosis when FSP1 is concurrently silenced or chemically blocked, confirming that the FSP1–CoQ_10_ axis compensates for GPX4 loss [78,79]. Under physiologic steady-state conditions, hematopoietic stem cells and common myeloid progenitors (CMPs) express minimal FSP1, yet its level rises sharply during myeloid differentiation [83]. Notably, FSP1 is preferentially upregulated in AML subtypes with recurrent genetic abnormalities, providing a molecularly defined therapeutic window [83]. Collectively, in ALL—characterized by negligible FSP1—GPX4 inhibition alone is sufficient to trigger ferroptosis, whereas dual targeting of GPX4 and FSP1 represents a rational strategy for high-FSP1 AML.

### 4.8. Prostate Cancer

The role of FSP1 in prostate cancer (PCa) is complex, with its expression showing significant heterogeneity across different disease stages and subtypes. While some studies report lower FSP1 levels in primary PCa tissues compared to benign prostate tissue, its expression is paradoxically and significantly elevated in high-risk subgroups, particularly those with advanced-stage disease [84,85,86]. Multiple ferroptosis-related prognostic models in prostate cancer have identified elevated FSP1 expression as a critical marker predicting unfavorable outcomes, correlating closely with higher Gleason scores (≥8), shorter biochemical recurrence intervals, and increased propensity for castration-resistant progression [85,87]. Moreover, preclinical studies in prostate cancer models have demonstrated that pharmacologic or genetic inhibition of FSP1 markedly restores tumor sensitivity to abiraterone, accompanied by activation of ferroptosis and accumulation of lipid-reactive oxygen species (ROS) [87,88]. Nevertheless, large-scale clinical data defining the precise proportion of prostate cancer patients characterized by high FSP1 expression remain unavailable, and robust evidence supporting a direct role of FSP1 in bone metastasis progression or radiotherapy resistance is still lacking [88]. These unresolved questions necessitate further clinical and experimental investigations.

### 4.9. Ovarian Cancer

High FSP1 expression significantly correlates with inferior overall survival (OS) and progression-free survival (PFS) in ovarian cancer, and predicts platinum resistance and early recurrence [89]. Poly(ADP-ribose) polymerase (PARP) inhibitors, such as olaparib and niraparib, have become standard first-line or maintenance therapies for advanced ovarian cancer, particularly in patients harboring BRCA1/2 mutations; however, their clinical benefit remains limited in BRCA-proficient tumors [90,91]. Recent evidence indicates that beyond its canonical anti-ferroptotic function, FSP1 physically interacts with Ku70 to facilitate DNA-PKcs-mediated non-homologous end-joining (NHEJ), thereby conferring resistance to PARP inhibitors [89]. Pharmacological or genetic inhibition of FSP1 markedly enhances PARP inhibitor-induced DNA damage via a ferroptosis-independent mechanism [89]. Moreover, a junctional adhesion molecule 3 (JAM3)-driven NRF2–FSP1 signaling axis has recently emerged as a critical mediator of dual resistance to cisplatin and ferroptosis, highlighting the potential of targeting FSP1 or its upstream regulators to effectively overcome multidrug resistance in ovarian cancer [25,92].

### 4.10. Brain Cancer

In malignant brain tumors, particularly glioblastoma and diffuse glioma, FSP1 expression is markedly elevated and consistently identified as a high-risk gene across multiple ferroptosis-based prognostic models, strongly implicating its role in poor clinical outcomes [93,94]. The expression of FSP1 in glioma is regulated at multiple levels. Psychological stress stabilizes FSP1 mRNA through the METTL3-mediated m^6^A modification, suppressing ferroptosis and promoting tumor progression [95]. Additionally, the recently identified lncRNA ryr3 divergent transcript (RYR3-DT) enhances lipid antioxidation via the CoQ_10_/FSP1 axis, further facilitating tumor invasiveness [96]. Pan-cancer analysis revealed that FSP1 expression positively correlates with ferroptosis resistance, and the NAD(P)H binding domain of FSP1 has been structurally validated as a promising druggable target. Therefore, developing FSP1 inhibitors or nano-delivery platforms capable of crossing the blood–brain barrier could represent an effective strategy to improve glioblastoma therapy [11,24]. Considering the critical implications of FSP1 across various cancers, it is essential to systematically summarize its distinct roles and targeted therapeutic strategies. To provide a clear overview, the expression and functional characteristics of FSP1 and underlying molecular pathways involving it in major cancer types are outlined in Table 1.

## 5. Conclusions

As an essential hub in the ferroptosis regulatory network, FSP1 leverages its unique CoQ10 and VK reductase activities to shield cancer cells from oxidative and ferroptotic damage, thereby fueling tumor progression and treatment resistance across diverse malignancies. Currently, multiple strategies targeting FSP1, such as specific small-molecule inhibitors, metabolic interventions, and combinational therapies with immunotherapy or chemotherapy, are undergoing rigorous preclinical evaluation, demonstrating encouraging therapeutic efficacy. Notably, emerging FSP1 inhibitors have shown potential to synergistically enhance sensitivity to ferroptosis induction when combined with GPX4 inhibitors or conventional anticancer therapies. Nonetheless, the therapeutic targeting of FSP1 remains in the preclinical research stage and faces multiple challenges, including drug selectivity, delivery efficiency, potential resistance mechanisms, and clinical translation. Addressing these hurdles will require further optimization of inhibitor potency and specificity, improved drug delivery systems, and comprehensive mechanistic studies.

Mounting evidence continues to support FSP1 inhibition as a promising strategy for surmounting therapeutic resistance and amplifying the effectiveness of current treatment regimens. Future efforts should focus on the clinical translation of FSP1 inhibitors through well-designed clinical trials, exploring their safety, pharmacokinetics, and efficacy profiles across diverse tumor types. With the continuous emergence of novel FSP1 inhibitors and further elucidation of their specific roles in different tumor types and disease stages, combined with precise molecular subtyping and personalized combination therapies, targeting FSP1 is expected to become a powerful addition to future oncological healthcare, offering new advancements in the management of treatment-resistant cancers.

## Figures and Tables

**Table 1:** Expression patterns, functional roles, and key mechanisms of FSP1 in various tumor types.

Tumor Type	Expression and Function of FSP1	Key Mechanisms	References
Hepatocellular Carcinoma	High expression; tumor-promoting	FSP1 confers resistance to ferroptosis through the NRF2, lncFAL/TRIM69, and circ0060467 pathways and promotes metastasis via the SIRT1/PGC-1α axis.	[6,26,27,38,43,44,46,47,48,49]
Colorectal Cancer	High expression; tumor-promoting	FSP1 is regulated by NAT10-mediated mRNA ac4C modification, enhancing EMT and resistance to ferroptosis.	[30,50,51,52,53,55,56]
Pancreatic Ductal Adenocarcinoma	High expression; tumor-promoting	KRAS mutations enhance FSP1 via MAPK/NRF2, promoting GPX4-mediated ferroptosis resistance and EMT-driven drug resistance.	[13,27,57,58,59,60,62,63]
Gastric Cancer	High expression; tumor-promoting	FSP1 and CISD1 co-upregulation promotes ferroptosis resistance and peritoneal metastasis via lipid droplet metabolism and tumor immune microenvironment modulation.	[40,64,65,66,67]
Breast Cancer	High expression; tumor-promoting	FSP1 maintains reduced CoQ10, driving oxidative stress resistance, TNBC progression, and drug resistance.	[7,11,68,69,70,71]
Lung Cancer	High expression; tumor-promoting	The KRAS/KEAP1/LKB1 pathway upregulates FSP1, mediating resistance to ferroptosis and radiotherapy via antioxidant defense.	[11,23,27,39,57,72,73,74,75,76,77,78]
Acute Lymphoblastic Leukemia	Low expression; tumor-suppressive	High promoter methylation silences FSP1, increasing cellular reliance on GPX4 to resist ferroptosis.	[79,80,81]
Acute Myeloid Leukemia	High expression; tumor-promoting	FSP1 and GPX4 are markedly co-upregulated in specific AML subtypes, representing potential precision therapy targets.	[82,83]
Prostate cancer	High expression; tumor-promoting	Elevated FSP1 expression promotes tumor progression by enhancing ferroptosis resistance, associated with advanced disease and poor prognosis.	[84,85,86,87,88]
Ovarian cancer	High expression; tumor-promoting	High FSP1 expression confers resistance to ferroptosis and PARP inhibitors through the NRF2–FSP1 signaling pathway and enhanced DNA repair capacity.	[25,89,90,91,92]
Brain cancer	High expression; tumor-promoting	Increased FSP1 expression driven by METTL3-mediated m6A modification and specific lncRNAs suppresses ferroptosis, facilitating aggressive tumor progression.	[93,94,95,96]

## Abbreviations

4-HNE	4-Hydroxynonenal
5-FU	5-Fluorouracil
ACLY	Atp-Citrate Lyase
AHR	Aryl Hydrocarbon Receptor
ALDH1A3	Aldehyde Dehydrogenase 1A3
ALL	Acute Lymphoblastic Leukemia
AML	Acute Myeloid Leukemia
AREs	Antioxidant Response Elements
CMPs	Common Myeloid Progenitors
CRC	Colorectal Cancer
CoQ10	Coenzyme Q10
CoQ10H2	Ubiquinol
DAMPs	Damage-Associated Molecular Patterns
EMT	Epithelial-To-Mesenchymal Transition
ERK	Extracellular Signal-Regulated Kinase
FAD	Flavin Adenine Dinucleotide
GC	Gastric Cancer
GCSCs	Gastric Cancer Stem Cells
GPD1	Glycerol-3-Phosphate Dehydrogenase 1
GPD1L	Glycerol-3-Phosphate Dehydrogenase 1-Like
GPX4	Glutathione Peroxidase 4
GSH	Glutathione
GSK3β	Glycogen Synthase Kinase 3 β
HCC	Hepatocellular Carcinoma
HDLBP	High-Density Lipoprotein-Binding Protein
IDA	Indole-3-Acrylic Acid
IDRs	Intrinsically Disordered Regions
JAM3	Junctional Adhesion Molecule 3
JunD	Jun D Proto-Oncogene
KEAP1	Kelch-Like Ech-Associated Protein 1
LKB1	Liver Kinase B1
LLPS	Liquid–Liquid Phase Separation
MAPK	Mitogen-Activated Protein Kinase
METTL3	Methyltransferase-Like 3
MitoNEET	Mitochondrial Iron–Sulfur Protein
NAD(P)H	Nicotinamide Adenine Dinucleotide (Phosphate)
NADH	Nicotinamide Adenine Dinucleotide
NAT10	N-Acetyltransferase 10
NHEJ	Non-Homologous End-Joining
NSCLC	Non-Small-Cell Lung Cancer
OS	Overall Survival
PARP	Poly (ADP-Ribose) Polymerase
PCa	Prostate Cancer
PDAC	Pancreatic Ductal Adenocarcinoma
PFS	Progression-Free Survival
PGC-1α	Peroxisome Proliferator-Activated Receptor Gamma Coactivator 1-Alpha
ROS	Reactive Oxygen Species
RYR3-DT	Ryr3 Divergent Transcript
SLC25A1	Solute Carrier Family 25 Member 1
SLC7A11	Solute Carrier Family 7 Member 11
TME	Tumor Microenvironment
TNBC	Triple-Negative Breast Cancer
TNM	Tumor–Node–Metastasis
TRIM21	Tripartite Motif Containing 21
TRIM54	Tripartite Motif Containing 54
TRIM69	Tripartite Motif Containing 69
USP7	Ubiquitin-Specific Protease 7
VK	Vitamin K
YTHDC1	YTH Domain Containing 1
YTHDF2	YTH Domain Family Member 2
ac4C	N4-Acetylcytidine
acetyl-CoA	Acetyl-Coenzyme A
circRNAs	Circular RNAs
iFSP1	Inhibitor Of Ferroptosis Suppressor Protein 1
icFSP1	Improved Chemical Inhibitor Of Ferroptosis Suppressor Protein 1
lncFAL	Ferroptosis-Associated Long Non-Coding RNA (lncFAL)
lncRNAs	Long Non-Coding RNAs
m6A	N6-Methyladenosine
mRNA	Messenger RNA
mTOR	Mechanistic Target Of Rapamycin
miR-4443	MicroRNA-4443
miR-6805	MicroRNA-6805
miRNA	MicroRNA
β-TrCP	β-transducin repeat-containing protein
